# Adolescents’ emotional competence is associated with parents’ neural sensitivity to emotions

**DOI:** 10.3389/fnhum.2014.00558

**Published:** 2014-07-23

**Authors:** Eva H. Telzer, Yang Qu, Diane Goldenberg, Andrew J. Fuligni, Adriana Galván, Matthew D. Lieberman

**Affiliations:** ^1^Department of Psychology, University of IllinoisUrbana-Champaign, IL, USA; ^2^Beckman Institute for Advanced Science and Technology, University of IllinoisUrbana-Champaign, IL, USA; ^3^Department of Psychology, University of CaliforniaLos Angeles, CA, USA; ^4^Department of Psychiatry and Biobehavioral Sciences, University of CaliforniaLos Angeles, CA, USA; ^5^Brain Research Institute, University of CaliforniaLos Angeles, CA, USA

**Keywords:** adolescence, emotional competence, family, fMRI, emotions

## Abstract

An essential component of youths’ successful development is learning to appropriately respond to emotions, including the ability to recognize, identify, and describe one’s feelings. Such emotional competence is thought to arise through the parent–child relationship. Yet, the mechanisms by which parents transmit emotional competence to their children are difficult to measure because they are often implicit, idiosyncratic, and not easily articulated by parents or children. In the current study, we used a multifaceted approach that went beyond self-report measures and examined whether parental neural sensitivity to emotions predicted their child’s emotional competence. Twenty-two adolescent–parent dyads completed an fMRI scan during which they labeled the emotional expressions of negatively valenced faces. Results indicate that parents who recruited the amygdala, VLPFC, and brain regions involved in mentalizing (i.e., inferring others’ emotional states) had adolescent children with greater emotional competence. These results held after controlling for parents’ self-reports of emotional expressivity and adolescents’ self-reports of the warmth and support of their parent relationships. In addition, adolescents recruited neural regions involved in mentalizing during affect labeling, which significantly mediated the associated between parental neural sensitivity and adolescents’ emotional competence, suggesting that youth are modeling or referencing their parents’ emotional profiles, thereby contributing to better emotional competence.

## INTRODUCTION

Competent emotional functioning is essential for well-being. The ability to recognize, identify, and describe one’s own and others’ feelings is considered key aspects to emotional competence. While emotional competence includes aspects of emotion regulation (Shipman and Zeman, 2001), for the purpose of this article, we define emotional competence as an understanding of ones own and others’ emotions and the ability to display emotions in a situationally appropriate manner (Eisenberg et al., 1999). Individuals who lack these skills are often characterized in terms of alexithymia, which is a marked inability to identify, describe, and express one’s emotions. Emotional competence contributes to the achievement of both intrapersonal (e.g., individual well-being) and interpersonal (e.g., maintenance of important social relationships) well-being (Eisenberg et al., 2001; Shipman and Zeman, 2001). Indeed, difficulties in identifying feelings and emotions underlie depression, anxiety, delinquency, and impaired friendships (Honkalampi et al., 2009). Better accuracy in interpreting others’ emotions relates to youths’ ability to successfully interact with their peers and is associated with better academic adjustment (Eisenberg et al., 1999; Halberstadt et al., 2001). In contrast, poor emotional competence can have negative implications for youths’ well-being, including difficulty forming friendships and poorer academic adjustment (Eisenberg et al., 1999).

The adolescent years mark an essential time to understand the development of emotional competence. Adolescents experience more frequent and intense emotions than younger or older individuals (Silk et al., 2003), perhaps due to neurobiological changes. Neural systems implicated in emotional processing go through a “social reorientation” during adolescence (Nelson et al., 2005). Moreover, the prevalence of various forms of psychopathology that are characterized by deficits in emotional processing (e.g., depression) increases dramatically during the adolescent period (Hankin et al., 1998). It is therefore essential to examine how youth develop emotional competence in order to better understand individual differences in adolescent mental health and adjustment.

There is converging evidence that youths’ emotional competence is shaped by their environment. Although many sources contribute to the development of emotional competence (e.g., family, school, peers, genetics), parents remain one of the most influential sources by which youth learn to label, identify, and interpret emotions (Halberstadt, 1991). Even for adolescents, most lessons about emotion come from the family (Naghavi and Redzuan, 2012). Despite decades of research indicating the importance of social contexts, particularly the family, there remains little understanding of the mechanisms by which social contexts impact youths’ emotional development (Morris et al., 2007).

Two primary mechanisms have been proposed by which parents transmit or promote the development of their child’s emotional competence. First, parental emotional expressivity is thought to promote children’s socioemotional competence. For example, parents who express many positive emotions and few negative emotions tend to have children with greater emotional competence (see Eisenberg et al., 2003). Moreover, parental emotional expressivity contributes to youths’ ability to interpret and understand others’ emotional reactions and to learn appropriate and effective emotional expressions in social interactions (Eisenberg et al., 2001). Indeed, in families with lower levels of expressivity, adolescents have higher alexithymia (Fukunishi and Paris, 2001). Secondly, parents who are warm and supportive may help their children to manage distress and cope successfully during emotionally arousing situations. In turn, adolescents in more warm and supportive homes develop better emotional competence (Eisenberg et al., 2003). For example, adolescents who report lower levels of family cohesion and support have higher alexithymia (Fukunishi and Paris, 2001; Karukivi et al., 2011).

Although these more explicit or direct mechanisms may be important, implicit and more indirect mechanisms likely play an important, and perhaps even more central role in adolescents’ emotional competence. Parents often unconsciously communicate emotions to their children through affect sharing and mirroring of affective expressions, which teach children how to label and interpret emotions and manage emotional arousal (Fukunishi and Paris, 2001). Such implicit methods of socialization contribute to shaping emotional competence in youth (Klimes-Dougan et al., 2007). Thus, parents’ own emotional experiences may teach children which situations provoke different emotions, which emotions are acceptable, how to manage the experience of those emotions, and appropriate reactions to emotions (Dunham et al., 1998; Eisenberg et al., 2003; Morris et al., 2007). When placed in a similar situation, children can then reference their parents’ emotional profiles, helping them to determine the affective meaning of the situation and react accordingly (Morris et al., 2007). This has been described as social referencing and modeling, which proposes that parents’ emotional profiles implicitly teach children which emotions are acceptable and expected in the family environment and how to manage the experience of emotions (Morris et al., 2007).

Despite the theorized importance of parents’ experience of emotions for their child’s emotional competence, the mechanisms through which parents share and transmit emotions have not been well characterized (Eisenberg et al., 2003). Because the transmission of emotions is often subtle, idiosyncratic, and not easily articulated by parents or children (Chaplin et al., 2005; Dunsmore et al., 2009), it is essential to use methodological tools that can go beyond the traditional self-report or behavioral observational approaches and assess more implicit forms of emotional experiences. Neuroimaging techniques are a key research tool used to measure the processing of emotions, which allows us to characterize parents’ emotional profiles without relying on self-report measures.

A number of brain regions have consistently been implicated in emotional processing. The amygdala is a limbic region that automatically detects emotional significance in one’s environment (Dolan and Vuilleumier, 2003; Liberzon et al., 2003). Damage to the amygdala produces deficits in identifying emotional expressions (Adolphs et al., 1995; Calder et al., 2000), highlighting the important role of this region in the ability to understand emotions. The ventrolateral PFC (VLPFC) is a region that is commonly activated during emotion recognition, active identification of emotion, and plays a central role in the regulation of emotional arousal (Wager et al., 2008). In addition, neural regions implicated in mentalizing (i.e., the ability to understand the mental states of others) are activated when inferring and understanding others’ emotional states, including the superior temporal sulcus (STS; de Gelder et al., 2004; Engell and Haxby, 2007; Grèzes et al., 2007; Peelen et al., 2007; Kreifelts et al., 2009), the dorsomedial PFC (DMPFC), temporal parietal junction (TPJ), temporal poles, and the precuneus/posterior cingulate cortex (PCC; Amodio and Frith, 2006; Mitchell et al., 2006; Saxe et al., 2006). Finally, the fusiform gyrus, and in particular the fusiform face area, is activated during visual processing of emotional faces (Vuilleumier et al., 2001; Ganel et al., 2005). Together, this network of brain regions signals the valance of emotions (amygdala), the understanding of emotional states (mentalizing network), the recognition of faces (fusiform), as well as the regulation of emotional arousal (VLPFC).

In the current study, we used self-report measures and fMRI to attain a multifaceted understanding of the processes by which parents transmit emotional competence to their adolescent child. We collected self-report measures of parents’ emotional expressivity and adolescents’ reports of parental warmth, two facets thought to be particularly important for the intergenerational transmission of emotional competence (Eisenberg et al., 2003). To capture parents’ emotional profiles that may not be articulated through self-reports, we examined parents’ emotional processing using fMRI. The ability to recognize, label, and understand the emotions of oneself and of other people is one of the most frequent and important forms of nonverbal emotional decoding (Lieberman, 2010) and underlies emotional competence. To tap this process, parents underwent a brain scan during which they completed an affect labeling task. By going beyond self-reports and scanning the brain during an emotion task, we can measure aspects of parents’ emotional processing that may occur outside of conscious awareness.

Our second goal was to examine the neural mechanisms in adolescents that may mediate the relationship between parental neural activation and adolescent emotional competence. Several researchers have proposed models by which the link between parental emotion-related behaviors and their children’s emotional competence is mediated by the ways in which youth respond to emotions themselves (Eisenberg et al., 2001, 2003; Morris et al., 2007). The ability to accurately receive others’ emotional communications is a central component of effective emotional competence (Halberstadt et al., 2001), and so youth may rely on referencing their parents’ emotional profiles when experiencing emotional arousal themselves. For example, Emde et al. (1991) posit that youth who are in an emotional situation can reference their parents’ emotion-related experiences, which allows the child to access internal depictions of parental emotion processes, thereby assisting them with emotion regulation and acting accordingly. Therefore, adolescents may recruit neural regions involved in mentalizing (i.e., inferring others’ emotional states) during affect labeling, suggesting that such youth are modeling or referencing their parents’ emotional profiles, thereby contributing to better emotional competence.

## MATERIALS AND METHODS

### PARTICIPANTS

Twenty-two parent–adolescent dyads (adolescents’ mean age = 17.71 years, 63% female) participated. All participants were from Mexican backgrounds and were currently residing in the Los Angeles metropolitan area. The parent who participated was the primary caregiver of the adolescent participant, which was defined as the parent who knew the child the most and spent the most time with the child. Nineteen of the parents were mothers and three were fathers. Twelve parents were currently married (i.e., both biological parents), three were separated or divorced, four were never married, and three were widowed. Participants completed written consent and assent in accordance with UCLA’s Institutional Review Board.

### ADOLESCENT QUESTIONNAIRE MEASURES

#### Emotional competence

Emotional competence was measured using the Toronto Alexithymia Scale (Bagby et al., 1994; Bach et al., 1996). Using a five-point scale (1 = “strongly disagree” to 5 = “strongly agree”), adolescents responded to 20 items examining (1) difficulty identifying feelings (e.g., “When I am upset, I don’t know if I am sad, frightened, or angry”); (2) difficulty describing feelings (e.g., “It is difficult for me to find the right words for my feelings”), and (3) externally oriented thinking (e.g., “I prefer to just let things happen rather than to understand why they turned out that way”). The 20 items were summed and reverse scored, such that higher scores indicated greater emotional competence.

#### Parental warmth and support

Family warmth and support was measured using the parent subscale of the Inventory of Parent and Peer Attachment (IPPA; Armsden and Greenberg, 1987). Using a five-point scale (1 = almost never to 5 = almost always), adolescents answered nine questions indicating to what extent they felt close to and supported by their parents. Example items include, “I could count on my parents when I needed to talk,” “My parents helped me to talk about my difficulties,” and “I trusted my parents.”

### PARENT QUESTIONNAIRE MEASURES

#### Berkeley Emotional Expressivity Questionnaire (BEQ)

The Berkeley Expressivity Questionnaire (BEQ; Gross and John, 1995) was administered to parents in order to examine parents’ self-report of their emotional expressivity. This 16-item measure assesses both the strength of emotional response tendencies and the degree to which these emotional impulses are expressed overtly. Items are scored on a seven-point Likert-type scale (1 = “strongly disagree” to 5 = “strongly agree”). The 16 items are averaged to create an index of self-reported parent emotional expressivity. In addition to the total score, the BEQ has three subscales. Negative Expressivity (e.g., “Whenever I feel negative emotions, people can easily see exactly what I am feeling”), Positive Expressivity (e.g., When I’m happy, my feelings show”), and Impulse Strength (e.g., “I have strong emotions”). Impulse Strength refers to intense emotional impulses that the individual finds difficult to control, whereas positive and negative expressivity refer to the expression of positive and negative emotions, respectively.

### fMRI PARADIGM

**FIGURE 1:**
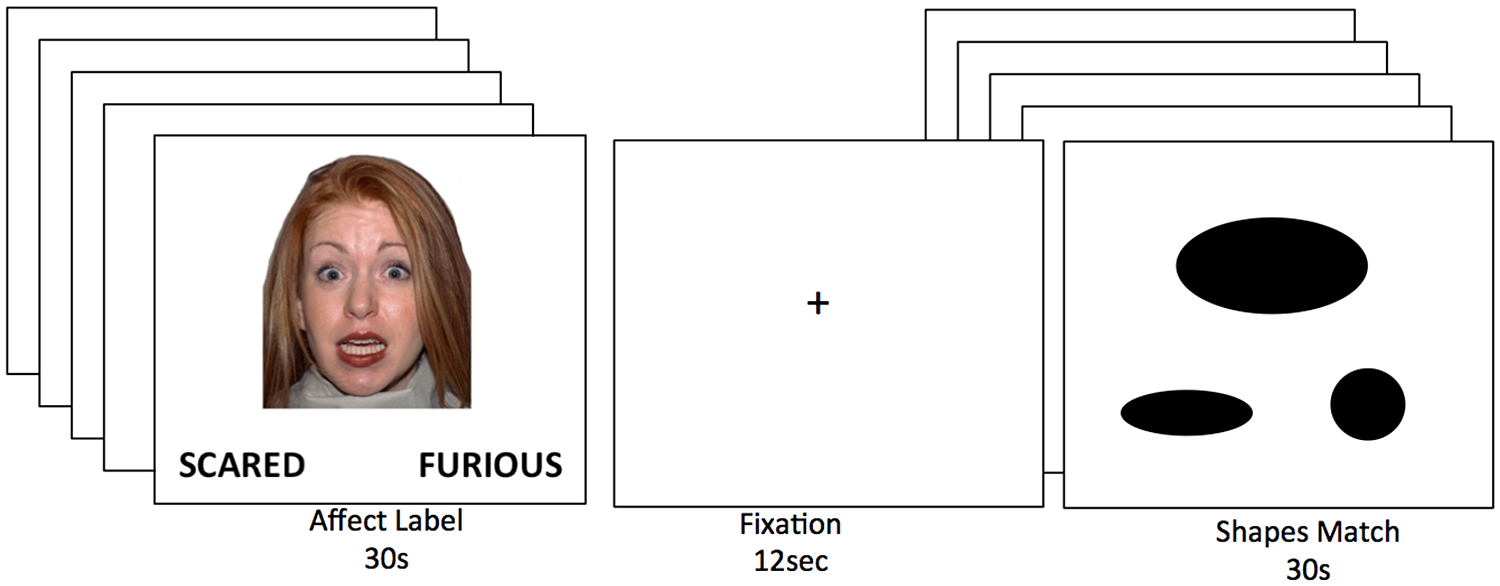
**The affect labeling task.** Conditions included two blocks of affect labeling and two blocks of shapes matching, separated by 12 s of fixation.

In order to measure neural processes involved when individuals label and process emotions, parents and adolescents completed an affect labeling task adapted from previous studies examining emotion matching and labeling (e.g., Hariri et al., 2000; Lieberman et al., 2007; Payer et al., 2011; Torrisi et al., 2013). During the task, participants were presented with a target face at the center of the screen and were instructed to choose the correct affect label (e.g., “scared,” “furious,” “angry,” “fearful,” “worried”) from a pair of words shown at the bottom of the screen (see **Figure 1**). Two affect labels were paired together, one with the target emotion and a second with a different negative emotion (e.g., scared – furious). The affect labels were selected from similar studies using this paradigm (e.g., Torrisi et al., 2013). Rather than matching faces (affect matching), as done in previous versions of this task (e.g., Hariri et al., 2000), the task condition involves verbal labeling of emotional facial expressions which more closely approximates the process of “putting feelings into words” (Lieberman et al., 2007). Target faces were male and female, and all facial expressions were depicting a negative emotion (fearful or angry). Half of the faces were African American and half were European American. Each block contained five 5-s trials and was preceded by 12-s of fixation. The faces remained on the screen for the entire 5-s trial. Participants completed two blocks of the emotional labeling task, interleaved with two blocks of a shape matching task (Hariri et al., 2000). The faces were selected from a standardized set of face stimuli (Tottenham et al., 2009).

### fMRI DATA ACQUISITION

Imaging data were collected using a 3 Tesla Siemens Trio MRI scanner. The task was presented on a computer screen, which was projected through scanner-compatible goggles. The affect labeling task consisted of T2*-weighted echoplanar images (EPI) [slice thickness, 4 mm; 34 slices; TR = 2 s; TE = 30 ms; flip angle = 90°; matrix = 64 × 64; FOV = 200 mm; voxel size 3 mm × 3 mm × 4 mm]. A T2*weighted, matched-bandwidth (MBW), high-resolution, anatomical scan and magnetization-prepared rapid-acquisition gradient echo (MPRAGE) scan were acquired for registration purposes (TR = 2.3 s; TE = 2.1 s; FOV = 256; matrix = 192 × 192; sagittal plane; slice thickness = 1 mm; 160 slices). The orientation for the MBW and EPI scans was oblique axial to maximize brain coverage.

### fMRI DATA PREPROCESSING AND ANALYSIS

Neuroimaging data were preprocessed and analyzed using Statistical Parametric Mapping (SPM8; Wellcome Department of Cognitive Neurology, Institute of Neurology, London, UK). Preprocessing for each participant’s images included spatial realignment to correct for head motion (no participant exceeded 2 mm of maximum image-to-image motion in any direction). The realigned functional data were coregistered to the high-resolution MPRAGE, which was then segmented into cerebrospinal fluid, gray matter, and white matter. The normalization transformation matrix from the segmentation step was then applied to the functional and structural images, thus transforming them into standard stereotactic space as defined by the Montreal Neurological Institute and the International Consortium for Brain Mapping. The normalized functional data were smoothed using an 8 mm Gaussian kernel, full width at half maximum, to increase the signal-to-noise ratio.

Whole-brain statistical analyses were performed using the general linear model in SPM8. Each trial was convolved with the canonical hemodynamic response function. High-pass temporal filtering with a cutoff of 128 s was applied to remove low-frequency drift in the time series. Serial autocorrelations were estimated with a restricted maximum likelihood algorithm with an autoregressive model order of 1. The task was modeled as a block design. The 12 s fixation prior to each block was not modeled and therefore served as an implicit baseline. The individual subject contrasts were submitted to random-effects, group-level analyses. To correct for multiple comparisons, we conducted a Monte Carlo simulation implemented using 3dClustSim in the software package AFNI (Ward, 2000). Results of 3dClustSim indicated a voxel-wise threshold of *p* < 0.005 combined with a minimum cluster size of 42 voxels for the whole brain, corresponding to *p* < 0.05, family wise error (FWE) corrected.

## RESULTS

### CORRELATIONS BETWEEN SELF-REPORT MEASURES

On average, adolescents’ emotional competence scores were 66.2 (SD = 11.12; range = 50–88). We ran bivariate correlations to examine how children’s emotional competence correlated with parental warmth and support and parental emotional expressivity. Emotional competence and parental emotional expressivity were not correlated (positive expressivity: *r* = 0.07, ns; negative expressivity: *r* = 0.22, ns; impulse strength: *r* = 0.01, ns). Emotional competence was associated with greater parental warmth and support, *r* = 0.49, *p* < 0.05. Parental emotional expressivity and parental warmth and support were not correlated.

### MAIN EFFECTS OF AFFECT LABELING TASK

#### Parental activation

**FIGURE 2:**
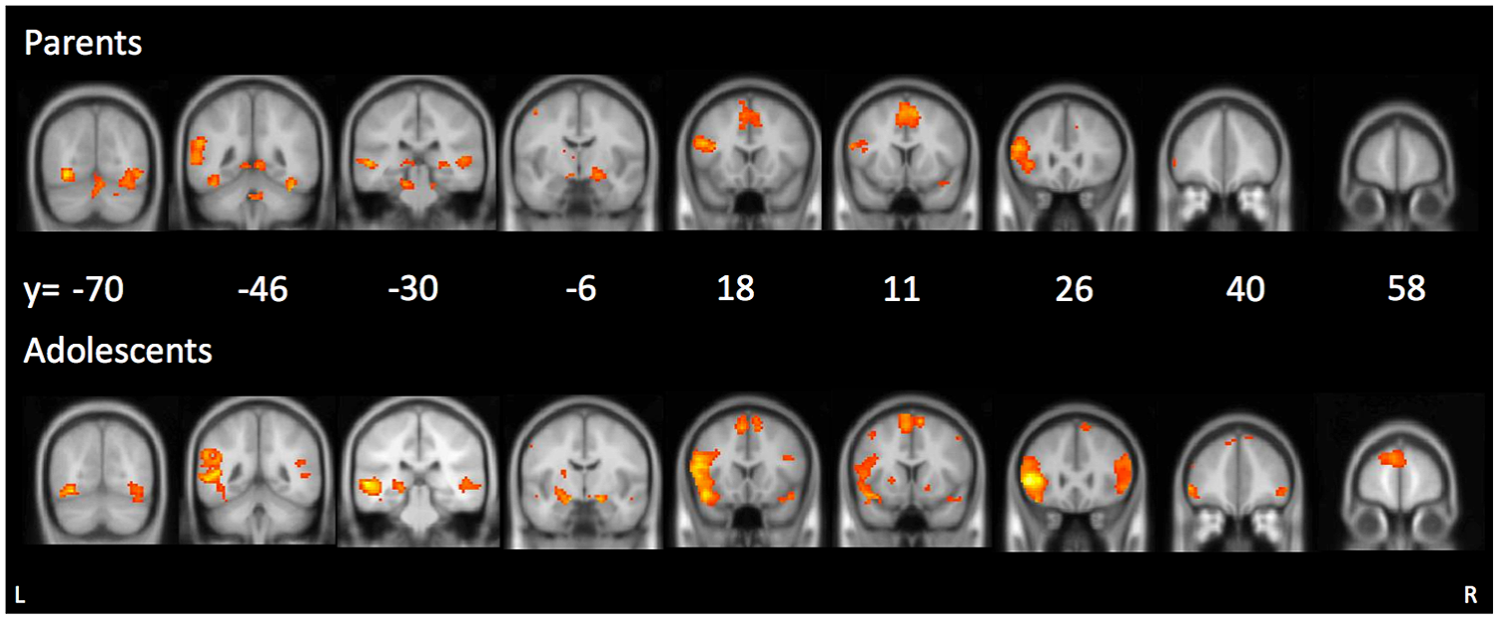
**Main effects during affect labeling for parents (top panel) and adolescents (bottom panel)**.

We first examined parents’ neural activation during affect labeling compared to shape matching. As shown in **Table 1** and **Figure 2**, parents demonstrated activation in the left VLPFC, bilateral pSTS, left TPJ, bilateral fusiform gyrus, right amygdala, precentral gyrus, and supplementary motor area.

**Table 1:** Neural regions activated during affect labeling versus shapes matching.

Anatomical region	BA	*x*	*y*	*z*	*t*	*k*
**Parents’ activation**
Left fusiform gyrus		-24	-82	-11	9.7	878
Right fusiform gyrus		30	-76	-17	5.5	1373^a^
Right amygdala		18	-7	-11	4.5	^a^
Left amygdala		-20	-13	-14	4.3	^a^
Left putamen		-9	2	-2	4.1	^a^
Lingual gyrus		9	-52	1	4.4	142
Left TPJ/pSTS		-57	-43	7	5.4	282
Right pSTS		51	-34	7	4.3	92
Right VLPFC	45/46/47	-48	35	1	4.7	401
Left precentral gyrus	6	-45	-1	43	4.6	113
Supplementary motor area	6	9	11	55	5.2	259
**Adolescents’ activation**
Left fusiform gyrus		-33	-70	-11	6.2	3122^a^
Right fusiform gyrus		42	-73	-17	4.0	^a^
Left pSTS		-51	-40	4	6.9	^a^
Left putamen		-21	5	-2	4.3	^a^
Left amygdala		-18	-4	-17	6.7	^a^
Right amygdala		18	-7	-14	5.5	77
Right pSTS		51	-34	4	4.1	254
Left VLPFC	45	-51	29	4	8.8	928
Right VLPFC	45	48	32	-2	5.9	332
DMPFC	8	3	50	46	3.8	67
DMPFC	10	-6	62	28	4.8	145
Right precentral gyrus	6	51	5	49	5.7	86
Left precentral gyrus	6	-42	2	46	5.5	104
Supplementary motor area	6	9	11	64	5.0	277

*x, y, and z refer to MNI coordinates; t, t-score at those coordinates (local maxima); BA, putative Brodman’s area; pSTS, posterior superior temporal sulcus; VLPFC, ventrolateral prefrontal cortex; SMA, supplementary motor area. All analyses were set at a threshold of p < 0.005, with 42 contiguous voxels, corresponding to FWE corrected. Regions that share the same superscript are part of the same cluster.*

#### Adolescent activation

We examined adolescents’ neural activation during affect labeling compared to shape matching. As shown in **Table 1** and **Figure 2**, adolescents demonstrated activation in the bilateral VLPFC, bilateral DLPFC, left caudate, bilateral amygdala, bilateral pSTS/TPJ, bilateral fusiform, DMPFC, and supplementary motor area. Notably, the neural regions activated in adolescents and parents during affect labeling were nearly similar. The only different brain region to emerge was the right amygdala and the right VLPFC in adolescents.

#### Adolescent versus parent activation

Although not a primary goal of the study, for exploratory purposes, we extracted parameter estimates of signal intensity from the regions of interest (ROIs) that showed a significant main effect in adolescents and parents during affect labeling versus shapes matching. We focused on the bilateral amygdala, left VLPFC, bilateral pSTS, and bilateral fusiform gyrus. In SPSS, we conducted paired samples *t*-tests to examine whether there were significant differences between parents and their adolescent child in neural response during affect labeling within these regions. No significant effects were found. Next, we ran correlation analyses to examine whether neural response in parents was correlated with neural response in their adolescent child. As shown in **Table 2**, there were no significant associations among the same regions (i.e., parent amygdala did not correlate with adolescent amygdala).

**Table 2:** Correlations between adolescent and parent neural responses during affect labeling versus shapes matching.

	Parent neural activation							
	Brain region	R amgy	L amyg	R VLPFC	R pSTS	L pSTS	R fusiform	L fusiform
**Adolescent neural activation**	R amyg	-0.24	-0.25	0.16	0.25	0.22	0.15	0.16
	L amyg	0.24	0.09	0.01	0.34	0.43*	0.15	-0.01
	R VLPFC	-0.21	0.31	0.29	0.21	0.41^†^	0.28	0.57**
	R pSTS	-0.52*	0.00	-0.05	0.23	0.05	0.13	0.19
	L pSTS	-0.03	0.35	0.25	0.36^†^	0.08	0.39^†^	0.33
	R fusiform	-0.23	0.08	-0.07	0.02	0.31	0.35	0.17
	L fusiform	-0.03	-0.02	-0.42*	0.27	0.27	0.11	-0.21

**p < 0.005; *p < 0.05; ^†^*p* < 0.10; *df* = 21. ROIs were extracted from the significant clusters described in **Table 1**. The ROIs were defined as a 6 mm sphere around the peak activation. pSTS, posterior superior temporal sulcus; VLPFC, ventrolateral prefrontal cortex; Amyg, Amygdala. R, right; L, left. To correct for the number of tests, a corrected *p* < 0.007 is needed for significance.

### NEURAL ACTIVATION AND ADOLESCENT EMOTIONAL COMPETENCE

#### Parental brain activation during affect labeling and adolescents’ emotional competence

**FIGURE 3:**
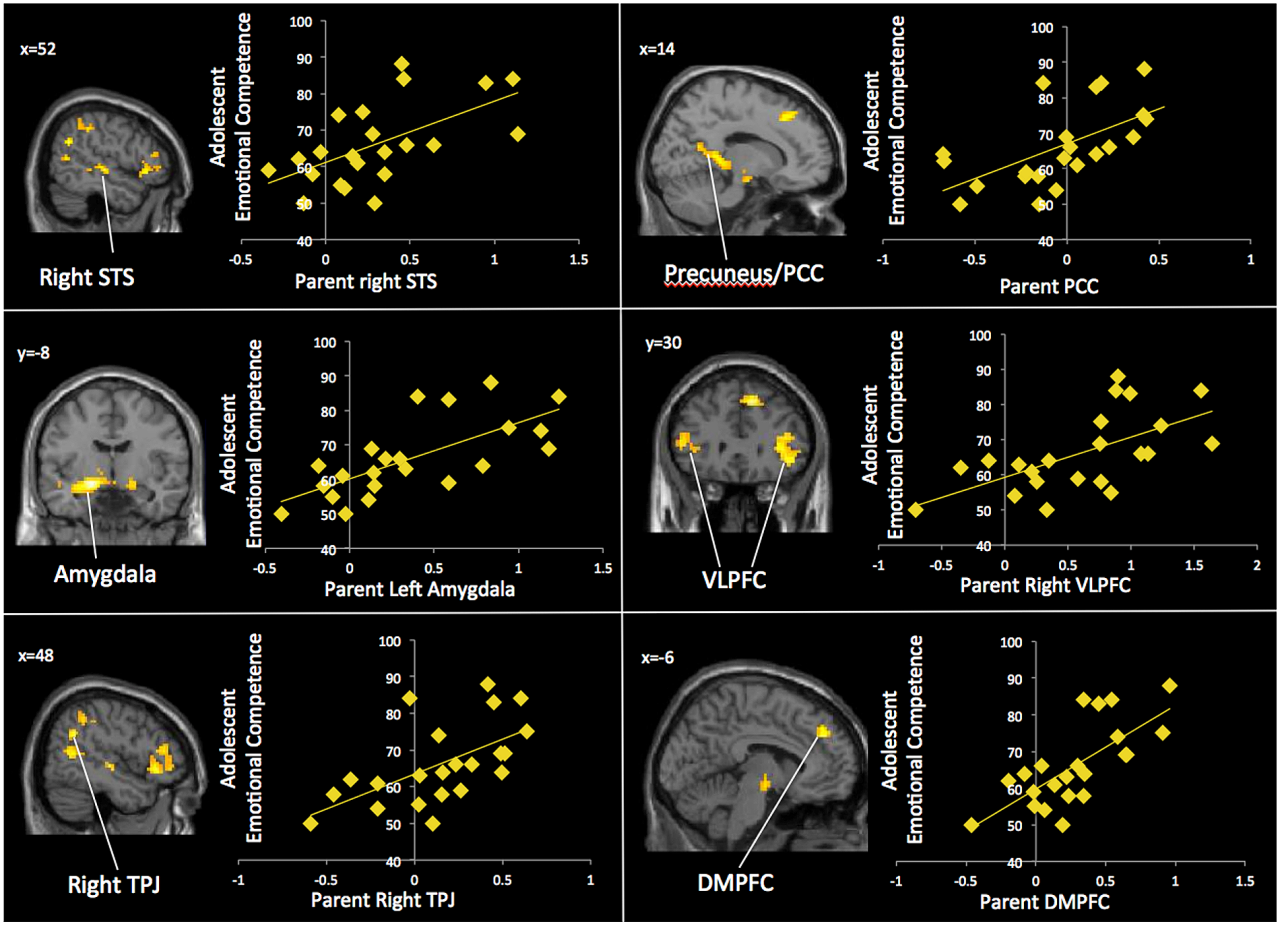
**Parents’ neural activation is associated with adolescents’ emotional competence**.

Next, we examined how parents’ neural activation was related to their adolescent child’s emotional competence. In whole-brain regression analyses, we entered adolescents’ emotional competence as a regressor to predict parents’ brain activation during affect labeling compared to the implicit baseline. As shown in **Table 3** and **Figure 3**, parents who evidenced greater activation in the bilateral amygdala, bilateral VLPFC, bilateral STS, right TPJ/pSTS, DMPFC, and precuneus had adolescents with greater emotional competence. Parents’ emotional expressivity scores and adolescents’ reports of parental warmth and support were entered as covariates in order to test whether brain activation relates to child emotional competence above and beyond self report of their emotional expressivity. All the clusters of activation remained significant. Parental emotion expressivity and adolescents’ report of parental warmth were not associated with neural activation during affect labeling. No brain regions correlated negatively with adolescents’ emotional competence. We correlated adolescents’ emotional competence with parents’ brain activation during shape matching compared to the implicit baseline. No brain regions were significantly correlated during shape matching.

**Table 3:** Neural regions activated in parents during affect labeling versus baseline that were positively associated with adolescents’ emotional competence.

	BA	*x*	*y*	*z*	*t*	*k*
Right amygdala		21	-7	-14	3.40	42
Left amygdala		-21	-4	-11	5.72	403
Left fusiform gyrus		-36	-40	-11	4.75	49
Right TPJ/pSTS		45	-52	10	4.52	70
Right TPJ		45	-55	31	4.32	178
Right STS		51	-19	-2	4.34	52
Left STS		-57	-19	-8	4.99	99
Right VLPFC	45/46/47	45	29	-8	4.45	253
Precuneus/PCC		15	-58	16	4.35	92
DMPFC	9	-6	41	34	4.67	44
DMPFC	8	12	29	46	5.02	91

*x, y, and z, MNI coordinates; t, t-score at those coordinates (local maxima); k, number of voxels in each significant cluster; BA, putative Brodman’s area; STS, superior temporal sulcus; VLPFC, ventrolateral prefrontal cortex; TPJ, temporal parietal junction; DMPFC, dorsomedial prefrontal cortex. All analyses were set at a threshold of *p* < 0.005, with 42 contiguous voxels, corresponding to FWE corrected.*

#### Adolescent brain activation during affect labeling and adolescents’ emotional competence

**FIGURE 4:**
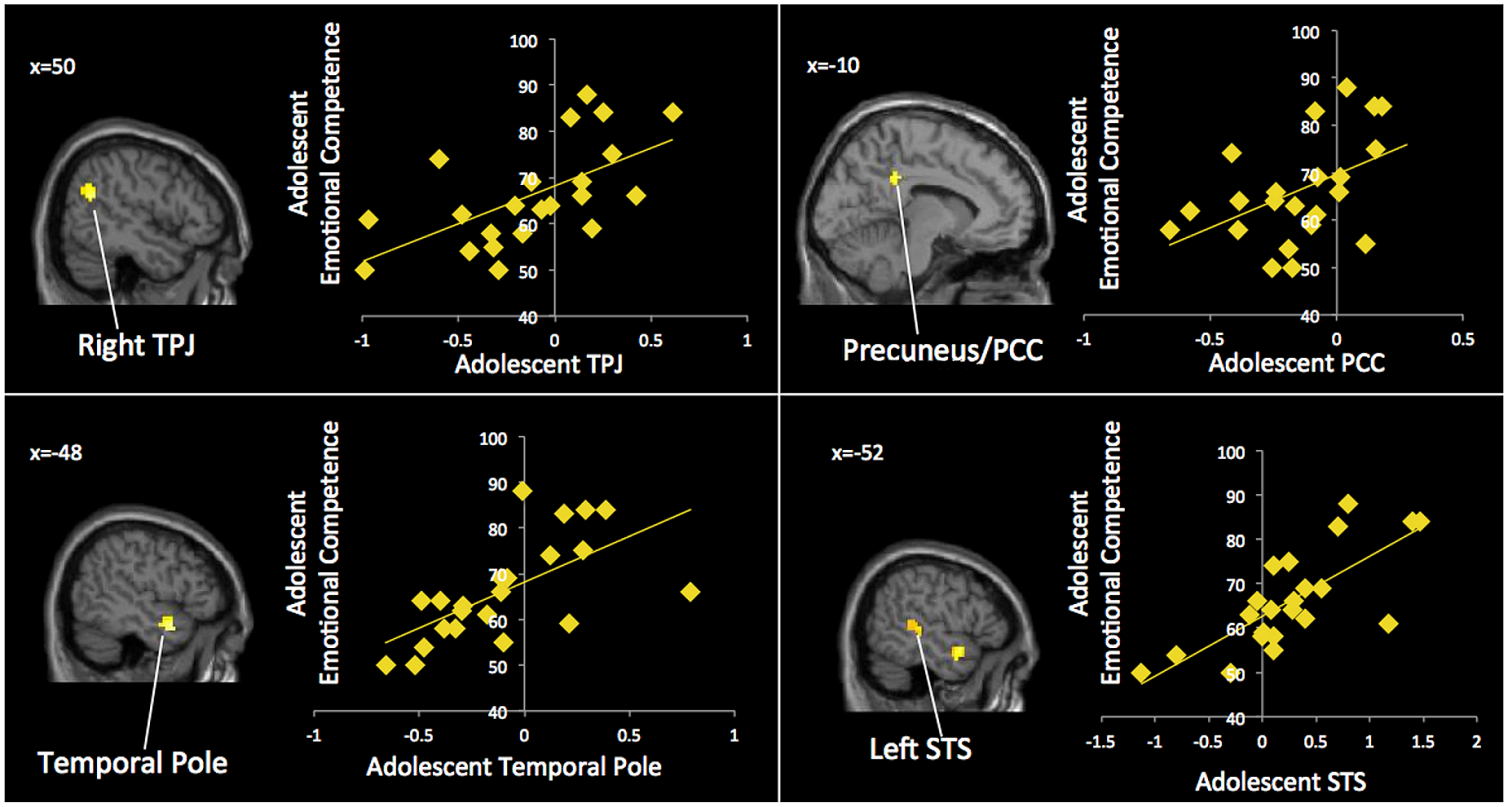
**Adolescents’ neural activation is associated with adolescents’ emotional competence**.

Our next set of analyses examined which neural regions in adolescents were associated with adolescents’ emotional competence. In whole-brain regression analyses, we entered adolescents’ emotional competence scores to predict brain activation during affect labeling compared to implicit baseline. As shown in **Table 4** and **Figure 4**, adolescents with greater emotional competence recruited the left STS, right TPJ, left temporal pole, PCC, and precentral gyrus to a greater extent during affect labeling. We found negative correlations in the cuneus and right DLPFC, suggesting that adolescents with poorer emotional competence recruit these regions to a greater extent during affect labeling.

**Table 4:** Neural regions activated in adolescents during affect labeling versus baseline that were associated with adolescents’ emotional competence.

Anatomical region	BA	*x*	*y*	*z*	*t*	*k*
**Positive correlations**
Right temporal pole	38	-45	5	-23	4.70	45
Right TPJ	39	52	-58	22	4.09	125
Left STS	21	-54	-31	4	4.37	86
Precuneus/PCC		-12	-37	28	4.02	83
Precentral gyrus	4	24	-25	55	4.01	47
**Negative correlations**
Cuneus	18	3	-79	22	4.40	102
Right DLPFC	9	36	35	41	4.94	152

*x, y, and z, MNI coordinates; t, t-score at those coordinates (local maxima); k, number of voxels in each significant cluster. BA, putative Brodman’s area. pSTS, posterior superior temporal sulcus; MPPC, medial posterior parietal cortex; DLPFC, dorsolateral prefrontal cortex. All analyses were set at a threshold of *p* < 0.005, with 42 contiguous voxels, corresponding to FWE corrected.*

#### Mediating adolescents’ brain activation on parental brain activation and adolescents’ emotional competence

Next, we tested whether adolescents’ neural activation during the same task mediated the association between parental brain activation and adolescents’ emotional competence. In other words, is the link between parental neural processing and adolescent emotional competence explained by the ways in which adolescents themselves respond to emotions? We first correlated the brain regions in parents and the brain regions in adolescents that were associated with adolescents’ emotional competence. We extracted parameter estimates of signal intensity from the significant clusters identified in the analyses above and ran correlation analyses in SPSS. As shown in **Table 5**, the majority of adolescents’ neural activations correlated with parents’ neural activations with the exception of the PCC which was largely uncorrelated for both adolescents and parents.

**Table 5:** Correlations between parent and adolescent neural responses in regions that correlated with adolescent emotional competence during affect labeling versus baseline.

		Parental neural activation					
		Amygdala	VLPFC	TPJ	pSTS	DMPFC	PCC
**Adolescent neural activation**	Temporal pole	0.52*	0.69***	0.51*	0.43*	0.38^†^	0.50*
	TPJ	0.54**	0.65***	0.73***	0.55**	0.51*	0.38^†^
	PCC	0.27	0.40^†^	0.41^†^	0.38^†^	0.34	0.33
	STS	0.45*	0.53*	0.41^†^	0.53*	0.47*	0.34

***p < 0.001; **p < 0.01; *p< 0.05; ^†^*p* < 0.10; *df* = 21. To correct for the number of tests, a corrected *p* < 0.008 is needed for significance.

For parsimony, we focused on the right TPJ for the mediation analyses. The right TPJ is the brain region most widely cited in studies of mentalizing and is selectively recruited during the attribution of mental states (Saxe and Wexler, 2005). A set of mediation analyses was performed in which the predicting variable was parental brain activation during affect labeling (e.g., amygdala), the outcome variable was the adolescents’ emotional competence score, and the proposed mediating variable was adolescents’ right TPJ activation. We controlled for parental emotional expressivity and warmth. As shown in **Table 6**, heightened parental brain activation during affect labeling (e.g., amygdala) was significantly predictive of their children’s greater right TPJ activation during the same task. When controlling for parental brain activation, heightened adolescent TPJ was significantly predictive of emotional competence. Using procedures described by Preacher and Hayes (2008), bootstrapping was performed, with 1000 samples and a bias-corrected confidence interval (CI) was created for the indirect effect. The indirect effect of parental brain activation on child emotional competence through adolescents’ neural response was significant for the 95% CI. Between 28 and 65% of the total effect was accounted for by adolescents’ TPJ activation. Similar mediation was found with adolescents’ temporal pole. Adolescents’ STS, PCC, and precentral gyrus did not significantly mediate any of the associations between parental brain response and adolescents’ emotional competence. For exploratory purposes, we ran mediation analyses with emotional competence as the mediator and adolescent brain activation as the outcome. Of the 11 mediation models, only two were significant. When adolescents’ TPJ was the mediator, all 11 models were significant, providing some confidence in the direction of the paths.

**Table 6:** Adolescent TPJ as a mediator of parental neural sensitivity and adolescent emotional competence.

Parent neural activation	Effect of IV on M (*a*)	Effect of M on DV (*b*)	Total effects (*c*)	Direct effects (*c*^′^)	% of total effect accounted for by mediators	95% CI
L amygdala	0.63**	0.32*	0.70***	0.50**	28.96	[0.32, 14.73]
R amygdala	0.72***	0.48*	0.55**	0.20	62.69	[1.23, 15.71]
L fusiform	0.72**	0.44*	0.66***	0.34	47.95	[0.72, 52.27]
R TPJ/pSTS	0.65**	0.36*	0.66***	0.43*	35.51	[0.12, 20.43]
R TPJ	0.53*	0.43*	0.61***	0.38*	37.73	[0.63, 17.48]
R STS	0.75***	0.50*	0.58**	0.20	65.48	[0.46, 30.89]
L STS	0.67**	0.39*	0.64***	0.38*	40.67	[0.29, 21.33]
R VLPFC	0.62**	0.37*	0.62***	0.39*	37.34	[0.15, 10.10]
Precuneus/PCC	0.52*	0.47**	0.61**	0.37*	39.37	[0.15, 16.77]
DMPFC	0.64**	0.41*	0.68***	0.42*	38.59	[0.24, 20.29]
DMPFC	0.58**	0.35*	0.66***	0.45**	30.90	[0.12, 19.01]

IV, independent variable. The independent variable represents parents’ neural activation and the mediator represents adolescents’ TPJ activation. Parents’ emotional expressivity and parental warmth were covariates. All coefficients reported are standardized. Bootstrapping was performed: 1000 samples were requested and a bias-corrected 95% confidence interval (CI) was created for the indirect effect. STS, superior temporal sulcus; VLPFC, ventrolateral prefrontal cortex; TPJ, temporal parietal junction; DMPFC, dorsomedial prefrontal cortex. ^†^*p* < 0.1; *p < 0.05; **p < 0.01; ***p < 0.001.

## DISCUSSION

An essential component of youths’ successful development is learning to appropriately respond to emotions in socially appropriate ways. Such emotional competence is thought to arise through the parent–child relationship (Eisenberg et al., 1998). Yet, the mechanisms by which parents transmit emotions are difficult to measure because they are often implicit, idiosyncratic, and not easily articulated by parents or children (Chaplin et al., 2005; Dunsmore et al., 2009). In the current study, we used a multifaceted approach that went beyond self-report measures and examined whether parental neural sensitivity to emotions predicted their child’s emotional competence above and beyond parents’ report of emotional expressivity and adolescents’ reports of the warmth and support of their parent relationships. We also examined whether adolescents’ neural responses to emotions mediated the link between parental neural sensitivity and adolescents’ emotional competence. Such a methodological approach allowed us to assess more implicit forms of emotional experiences.

Individual differences in the recruitment of parents’ amygdala, VLPFC, and several mentalizing regions were associated with their adolescent child’s emotional competence, suggesting that the ways that parents respond to emotions has an impact on their child’s emotional competence. Heightened amygdala reactivity could suggest greater engagement in the encoding of emotional stimuli, greater emotional responses, enhanced emotional awareness, or more recruitment of attentional resources, all of which can bring emotional stimuli to the forefront of awareness (Kugel et al., 2008). Thus, parents who demonstrate heightened amygdala response when identifying emotions in their environment may implicitly communicate to children which emotions to attend to. In contrast, parents with reduced amygdala reactivity may have an attenuation of basic emotional experiences, which may contribute to problems in identifying and differentiating feelings (Reker et al., 2010), and so these parents have children with lower emotional competence. Parents’ lateral PFC activation was also associated with adolescents’ emotional competence. The VLPFC has consistently been linked with effective emotion regulation (Wager et al., 2008) and is suggested to be particularly important for dampening emotional arousal when labeling emotional states, or “putting feelings into words” (Lieberman et al., 2007). Therefore, VLPFC activation may be associated with subtle external cues on how to effectively regulate emotions. Finally, greater recruitment of brain regions involved in mentalizing may suggest parents engage in greater perspective taking, which could benefit the child’s emotional competence.

We also examined the neural mechanisms in adolescents that are associated with their emotional competence. We found that adolescents with greater emotional competence showed heightened activation specifically in regions involved in mentalizing, including the TPJ, STS, temporal pole, and MPPC. The TPJ in particular may contribute to emotional insight, allowing adolescents to reference their parents’ emotional experiences during emotion processing. Adolescents with higher emotional competence may be relying on their ability to mentalize and think about their parents’ reactions to emotions. In contrast, lower levels of activation in regions involved in mentalizing may contribute to poorer emotional competence by impairing or limiting adolescents’ emotional insight. Adolescents may learn to regulate their emotions through modeling parents’ responses to emotions (Morris et al., 2007), as well as referencing parents’ emotional expressions and behavior (e.g., Eisenberg et al., 1996, 1999; Klimes-Dougan et al., 2007). Indeed, mediation analyses confirmed that parents’ neural processing significantly predicted adolescents’ neural processing specifically in brain regions involved in mentalizing, which, in turn, was associated with adolescents’ emotional competence. Thus, adolescents may access internal depictions of parental emotion processes, thereby assisting them with interpreting and understanding emotions and contributing to their emotional competence. Our findings are consistent with theoretical models suggesting that the link between parental emotion-related behaviors and their children’s emotional competence is mediated by the ways in which youth respond to emotions themselves (Eisenberg et al., 2001, 2003; Morris et al., 2007). However, in interpreting these mediation analyses, one should be cautious, as we did not use longitudinal measurements in order to carefully map the trajectory of these associations. It is possible that the effects are bidirectional and that adolescents’ emotional competence affects the way they respond at the neural level to emotions.

The current study used a unique methodological technique, whereby we used parental neural responses to emotion to predict adolescents’ emotional competence. The ways in which parents responded at the neural level during affect labeling was associated with their child’s emotional competence above and beyond parents’ reports of their emotional expressivity and adolescents’ reports of parental warmth and support. These findings underscore the value of using fMRI to tap more implicit processes that may not be measurable with traditional self-reports. This study is significant in light of a growing trend in neuroimaging research to move beyond brain mapping and statistical association to actual prediction of behavior and well-being (Falk et al., 2010). For example, researchers have begun to use neural activation to predict behavior and well-being either concurrently (Haxby et al., 2001) or in the future (Soon et al., 2008; Falk et al., 2010, 2011; Masten et al., 2011; Telzer et al., 2013), allowing researchers to examine whether there are neural predictors of behaviors and feelings. We build upon these advances by examining the neural markers in parents that may predict their child’s emotional experiences.

Perhaps the most unique contribution in the current study was our use of scanning both parents and their adolescent child. To our knowledge, no prior study has reported at the same time results of scanning parent–child dyads on the same task and relating their brain function. By scanning parent–child dyads, we were able to capture a more complex process whereby parents’ neural processing of emotions contributes to adolescents’ neural processing of emotions, thereby contributing to adolescents’ emotional competence. Researchers have suggested that parents’ emotion-related experiences and their children’s emotional competence is mediated by the ways in which youth respond to emotions themselves (Eisenberg et al., 2001, 2003). By scanning both parents and their child, we were able to demonstrate this meditational process. Future studies should scan pairs of individuals to continue to unwrap the mechanisms by which emotions are shared between individuals.

While parent–child socialization of emotions is proposed to be a major contributor of adolescents’ development of emotional competence, other mechanisms can also be at place. For example, peers become increasingly salient socializers during the adolescent years and may contribute to adolescents’ understanding of emotions. As adolescents spend more time with their peers, they may redirect their attention to the ways in which their peers process emotions. Secondly, genetics may account for some of the associations that we found. It is possible that shared genes account both for parents’ neural responses to emotions and youths’ emotional competence, and socialization in itself may not be as important. It is also likely that the transmission of emotion is reciprocal. Although in the current study we test the link from parent’s emotional profiles to children’s emotional competence, the dynamics between parents and children are bidirectional. Children’s emotional disposition (i.e., temperament), for example, can influence how parents respond to and experience emotions, which in turn can have effects on the child (Zahn-Waxler, 2010). Similarly, children with different temperaments may benefit differently from their parents’ emotional experiences. For example, children high in negative reactivity stand to benefit the most from parenting that supports refining and understanding of emotions (Morris et al., 2007). It is important for future research to further examine the reciprocal influences of emotion experiences within families.

It is important to note that the majority of parents in the current study were mothers. While mothers have been noted to be the “emotional gatekeeper” of the family, fathers are very important contributors as well (Denham et al., in press). Due to cultural gender roles, mothers and fathers may experience emotions differently, with fathers tending to inhibit their emotional experiences more (Birnbaum and Croll, 1984). These gender roles are transmitted to children, such that parents may socialize emotions to their daughters and sons in different ways. For example, social learning theory (Bandura and Walters, 1963) suggests that social referencing may depend on gender, such that parenting behaviors have a stronger effect on same-sex than opposite-sex children (Fabes et al., 1990). Moreover, significant research has found that children’s gender influences their parents’ emotion socialization efforts, with girls receiving more affective support than boys (Fabes et al., 1994). Future research should examine the differential roles of mothers and fathers, as well as gender differences among adolescents, in order to better capture the intergenerational transmission of emotions.

Emotion socialization processes are embedded within culture. Culture affects how parents interact with their children, the emotional climate of the family, and the ways in which emotions are interpreted and expressed (Morris et al., 2007). These distinct cultural norms will shape emotion socialization practices. Furthermore, the same parenting behaviors may have different effects on youth from different cultures, such that parenting behaviors hold a different psychological meaning across cultures. Thus, the findings in the current study may not extend to families from other ethnic/cultural groups. Future research is needed that delineates these complex processes across cultural groups in order to explore cultural differences in the intergenerational transmission of emotions and the associated child outcomes.

Although not a key goal of the study, it is interesting to note the similarities and differences in neural profiles among adolescents and parents. When we examined the correlation between parents and adolescents for brain regions that were associated with emotional competence, we found high levels of parent–child overlap (i.e., strong correlations). For example, adolescents’ TPJ was highly correlated with parents’ TPJ activation. Importantly, not all regions in adolescents correlated with parental activation. For example, the brain regions that were activated for adolescents and parents in the main effects showed minimal overlap (i.e., weak and non-significant correlations). This suggests specificity in child–parent neural patterns and rules out the possibility that BOLD responses merely correlate within family members due to shared physiological responses. If this were the case, brain responses in parents should correlate with their adolescent child’s brain responses. Our findings suggest that the level of correspondence between parents and adolescents depends in part on youths’ emotional competence. In other words, only regions that were significantly associated with emotional competence in adolescents and parents were also correlated with one another (e.g., high correlations between parent and adolescent in TPJ). Future research should carefully examine neural coupling within parent–child dyads.

In conclusion, we identify a potential neural mechanism by which adolescents develop effective emotional well-being. We examined the emotional processing of parents and their child by going beyond self-report measures and capturing a more nuanced and multifaceted understanding of the ways in which adolescents become emotionally competent. Future research should continue to examine the complex ways in which social contexts impact youths’ emotional development.

## Conflict of Interest Statement

The authors declare that the research was conducted in the absence of any commercial or financial relationships that could be construed as a potential conflict of interest.
